# Prognostic significance of CKAP2L expression in patients with clear cell renal cell carcinoma

**DOI:** 10.3389/fgene.2022.873884

**Published:** 2023-01-09

**Authors:** Zhi Liu, Jun Zhang, Deyun Shen, Xuechun Hu, Zongpan Ke, I Nyoman Ehrich Lister, Bungaran Sihombing

**Affiliations:** ^1^ Department of Urology Surgery, The First Affiliated Hospital of USTC, Division of Life Sciences and Medicine, University of Science and Technology of China, Hefei, Anhui, China; ^2^ Department of Urinary Surgery, The 901st Hospital of the Joint Logistics Support Force of PLA, Hefei, Anhui, China; ^3^ Universitas Prima Indonesia (UNPRI), Medan, North Sumatra, Indonesia

**Keywords:** ccRCC, CKAP2L, biomarker, prognostic model, immune infiltration

## Abstract

**Background:** Cytoskeleton-associated protein 2-like protein (CKAP2L) is thought to promote the progression of glioma, breast cancer, and ovarian cancer. However, the role of cytoskeleton-associated protein 2-like protein in clear cell renal cell carcinoma (ccRCC) is still unclear. The study aimed to investigate the roles and mechanisms of cytoskeleton-associated protein 2-like protein in clear cell renal cell carcinoma.

**Methods:** The level of cytoskeleton-associated protein 2-like protein in tumors was explored by using UALCAN and Oncomine databases. Gene expression datasets of clear cell renal cell carcinoma from The Cancer Genome Atlas and Gene Expression Omnibus (GEO) were also used to validate the cytoskeleton-associated protein 2-like protein level in clear cell renal cell carcinoma. Survival analysis was performed to investigate the relationship between cytoskeleton-associated protein 2-like protein level and prognosis of clear cell renal cell carcinoma patients. Cox regression analysis was used for identifying the independent prognostic factors. Gene set enrichment analysis (GSEA), gene set variation analysis (GSVA), protein–protein interaction analysis, co-expression analysis, and immune infiltration analysis were used to explore the potential mechanisms of cytoskeleton-associated protein 2-like protein in clear cell renal cell carcinoma. Moreover, the levels of cytoskeleton-associated protein 2-like protein in clinical clear cell renal cell carcinoma tissues were also measured using RT-PCR, immunohistochemical analysis, and Western blotting. M1 macrophages and CD4^+^ T cells were also detected by immunohistochemistry between tumor and normal tissues.

**Results:** The level of cytoskeleton-associated protein 2-like protein was upregulated in clear cell renal cell carcinoma according to multiple databases and experimental verification. Upregulated cytoskeleton-associated protein 2-like protein is an independent prognostic factor, which might activate the JAK–STAT signaling pathway, the P53 signaling pathway, the TGF-β signaling pathway, the WNT signaling pathway, *etc.*, in clear cell renal cell carcinoma. Protein–protein interaction analysis and co-expression analysis suggest that cytoskeleton-associated protein 2-like protein might interact with some proliferation proteins. Immune infiltration analysis indicates that cytoskeleton-associated protein 2-like protein may affect the level of activated CD4^+^ memory T cells, M1 macrophages, CD8^+^ T cells, and neutrophils in clear cell renal cell carcinoma. More M1 macrophage infiltrations in tumor tissues with higher cytoskeleton-associated protein 2-like protein were validated by clear cell renal cell carcinoma tumor tissues.

**Conclusion:** Cytoskeleton-associated protein 2-like protein is upregulated in clear cell renal cell carcinoma tissues, which may promote progression of the disease. Cytoskeleton-associated protein 2-like protein is a potential target for prognostic markers and a potential treatment target in clear cell renal cell carcinoma.

## Introduction

Clear cell renal cell carcinoma (ccRCC) prevails as a form of solid malignant tumor in the renal system (Ricketts and Linehan, 2018; Clark et al., 2019). In recent years, surgeons, physicians, and researchers have collaborated to enhance the use of surgical techniques and novel chemotherapeutic interventions (Bibok et al., 2021). However, complexities in local relapse and in distant metastasis are inevitable for some ccRCC patients, which seriously affects their survival interval and prognoses (Spiliopoulos et al., 2021). Therefore, it is very necessary to explore novel molecular markers and therapeutic targets for predicting the clinical outcome of ccRCC patients, preventing disease progression, and even completely curing ccRCC.

Cytoskeleton-associated protein 2-like protein (CKAP2L) is considered important for neural stem or progenitor cells. It is encoded by the CKAP2L gene, and it is usually referred as mitotic spindle protein (Yumoto et al., 2013). However, mutations in this gene are linked to spindle organization defects, such as mitotic spindle defects, lagging chromosomes, and chromatin bridges (Hussain et al., 2014; Capecchi et al., 2018). This is evident from the fact that mutations in this gene are linked with Filippi syndrome, in which symptoms such as growth defects, microcephaly, intellectual disability, facial feature defects, and syndactyly are developed (Karakaya et al., 2021). In addition, CKAP2L has been reported to be associated with disease progression and prognosis in a variety of tumors, including non-small-cell lung cancer, glioblastoma, hepatocellular carcinoma, and colorectal cancer (Xiong et al., 2019; Li et al., 2020; Wang and He, 2020; Feng et al., 2021). Nonetheless, the progress of CKAP2L in ccRCC is still unclear and needs to be elucidated.

In the present study, we first examined the expression of CKAP2L in multiple tumors, including ccRCC. Then, we explored the relationship between CKAP2L and a series of clinical characteristics and prognosis. We also investigated the potential effects of CKAP2L in ccRCC. Finally, we explored the relationship between CKAP2L and ccRCC immune microenvironments. Our study has revealed the crucial functions of CKAP2L in ccRCC. CKAP2L may provide a specific and helpful therapeutic target in ccRCC patients.

## Materials and methods

### Data access

The Cancer Genome Atlas (TCGA), kidney renal clear cell carcinoma (KIRC) expression data, and clinical phenotype were downloaded and preprocessed *via* GDCRNATools (Li et al., 2018). CEL files GSE76351, GSE53757, and GSE66270 were downloaded from the gene expression omnibus (GEO) database (https://www.ncbi.nlm.nih.gov/geo/). Microarray data were read from the CEL files and normalized *via* R packages affy and oligo, respectively. CKAP2L expression data in multiple tumors were accessed from the UALCAN database (http://ualcan.path.uab.edu/) and the Oncomine database (https://www.oncomine.com/) (Rhodes et al., 2004; Chandrashekar et al., 2017).

### Differential expression analysis

For ccRCC data from TCGA, differential expression analysis was performed with GDCRNATools using the DESeq2 method. The paired differential analysis was performed by using the DESeq2 package (Love et al., 2014; Li et al., 2018). For microarray data, limma was used for differential analysis. The log fold change >2 or < -2 and a *p*-value <0.05 were considered significant (Ritchie et al., 2015).

### Survival analysis

Overall survival (OS)/disease-free survival (DFS) and progression-free interval (PFI) used for survival analysis were downloaded from the UCSC XENA platform (https://xena.ucsc.edu/) (Goldman et al., 2020). Survival analysis was performed by the survival package. It is worth mentioning that OS and DFS data are in perfect agreement according to the UCSC XENA database. So, OS and DFS were combined and termed “OS/DFS” in our study.

### Cox regression analysis

Univariate and multivariate Cox regression analyses were performed by using the survival package. The CKAP2L level and clinical variables, including age, gender, tumor stage, and pathologic T and M stages, were used for univariate Cox regression analysis. Only significant variables were used for multivariate Cox regression. The pathologic M stage was not used for Cox regression because of its large deletions.

### Gene set enrichment analysis (GSEA) and gene set variation analysis (GSVA)

GSEA and GSVA were performed *via* clusterProfiler and GSVA packages, respectively (Hänzelmann et al., 2013; Wu et al., 2021). Results of differential gene expression analysis were used for gene ranking before GSEA. For GSVA, gene set score matrices were divided into higher- and lower-CKAP2L groups, and differential analysis was performed using the limma package. Only a *p*-value <.05 was considered significant.

### Immune infiltration analysis of ccRCC

The CIBERSORT algorithm was used for estimating the relative level of immune cells in each ccRCC sample from TCGA (Newman et al., 2015). Only the samples with less than .05 *p*-value were used for further analysis.

### Patients

This study included CKAP2L expression data from 10 patients who underwent radical nephrectomy at the Department of Urology at the First Affiliated Hospital of the University of Science and Technology of China between January 2020 and December 2020. This study was performed in agreement with the Declaration of Helsinki. All patients signed informed consent forms, and the procedures of our study were approved by the Ethics Committee of The First Affiliated Hospital of the University of Science and Technology of China. The present study was performed in agreement with the Declaration of Helsinki. Prior to surgery, every patient had not developed symptoms of distant metastases. However, patients with the following situations were not included in the study (Clark et al., 2019): those patients who had previously been treated with preoperative neoadjuvant therapy (radiation or chemotherapy) (Ricketts and Linehan, 2018); those patients who had a history of chromophobe renal cell carcinoma or papillary cell carcinoma; and (Bibok et al., 2021) patients lost to follow-up.

### Real-time polymerase chain reaction (RT-PCR)

Total RNA was isolated using TRIzol (15596-026, Invitrogen). cDNA was synthesized using 500 ng of total RNA using the PrimeScriptTM RT Reagent Kit (DRR037A; TaKaRa) according to the manufacturer’s instructions. RT-PCR was performed using SYBR premix EX Taq (TaKaRa) and analyzed with the StepOnePlus real-time PCR system (Thermo Fisher Scientific). The RT-PCR results, recorded as threshold cycle (Ct) numbers, were normalized against an internal control (GAPDH). The expression data were analyzed using the 2^−ΔΔCT^ method. The CKAP2L primer sequences were Forward: 5′-GAG​CCA​AAA​CAC​CAA​GCC​TTA-3′ and Reverse: 5′-GGA​GTT​TAA​TGC​TGA​TGG​ACC​TT-3′.

### Immunohistochemistry (IHC)

The samples of ccRCC were embedded in paraffin and cut into 4-μm sections before immunohistochemical staining following the standard protocol. Two expert uropathologists analyzed the complete results of the IHC separately. To avoid confusion, the uropathologists made use of a dual-headed microscope for analyzing and evaluating the results. The CKAP2L levels in sections of 10 patients mentioned previously were all detected. M1 macrophages and CD4^+^ T cells were also determined by IHC to evaluate the immune cell infiltration.

### Western blot

In the radioimmunoprecipitation assay (RIPA) buffer filled with the protease inhibitor mixture, tissues were collected followed by their lysates, and by means of bicinchoninic acid (BCA) assay, the concentration of total protein was measured. Subsequently, the wells of SDS-PAGE gel were used for loading the entire protein. However, after undergoing the process of electrophoresis, the overall protein was transferred onto PVDF membrane (Millipore, Billerica, MA, United States), thereby incubating the membrane in primary antibodies directed against CKAP2L (NBP1-83450, Novus Biologicals) and GAPDH (ab8245, Abcam). For combining primary antibodies, a horseradish peroxidase-conjugated secondary antibody (Vazyme, Piscataway, NJ, United States) was utilized, after washing with TBST buffer. At the end, the membrane with antibodies was visualized using the standard chemical luminescence method, and GAPDH was used for controlling the loading.

## Results

### CKAP2L is highly expressed in diverse tumors

**FIGURE 1:**
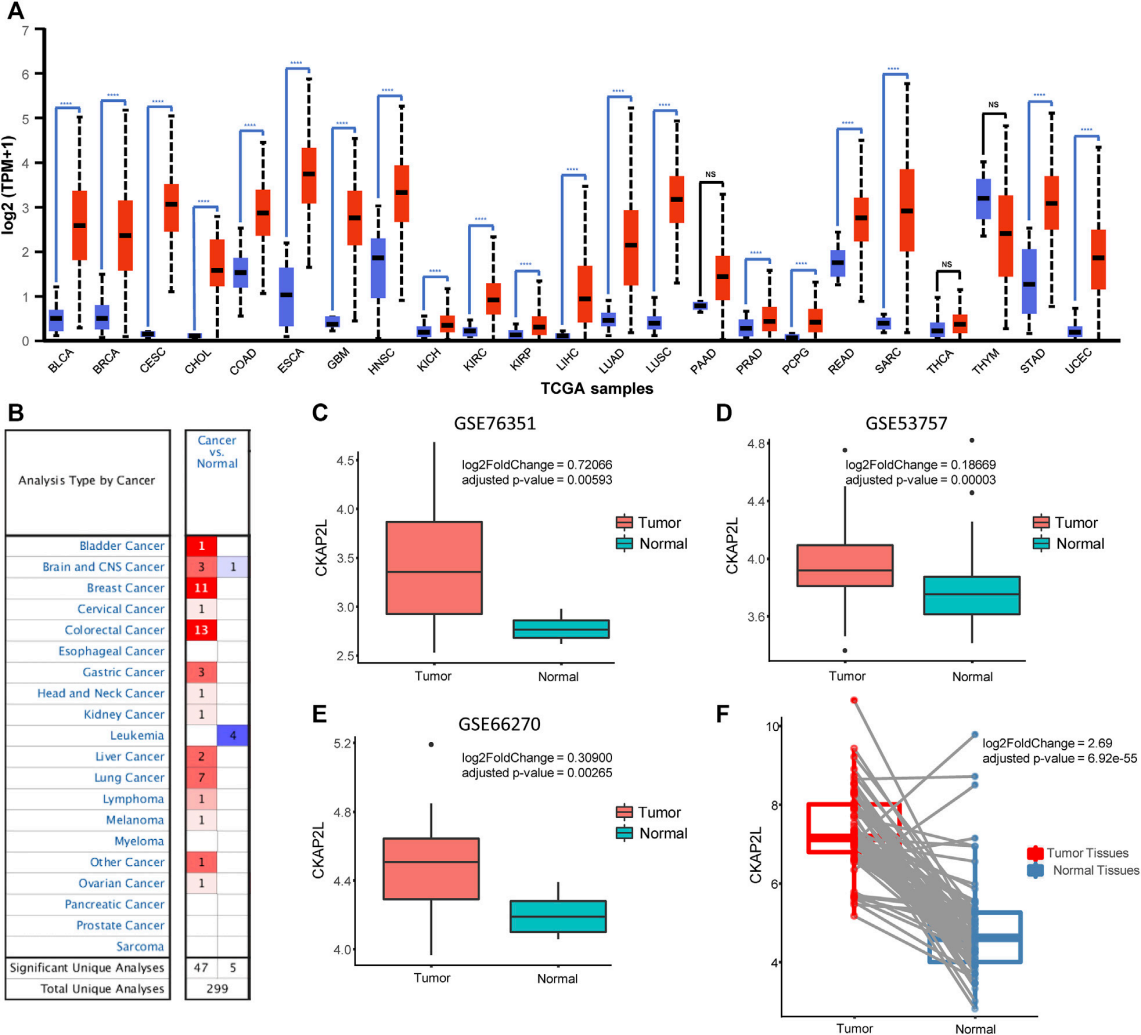
Cytoskeleton-associated protein 2-like protein (CKAP2L) is highly expressed in multiple tumor tissues. **(A)** Data from the UALCAN database indicated that CKAP2L is expressed at high levels in a variety of tumor tissues including clear cell renal cell carcinoma (ccRCC). **(B)** Oncomine database suggested that CKAP2L levels in multiple tumor tissues are higher than those in normal tissues. **(C–E)** Three datasets from the Gene Expression Omnibus (GEO) database imply that CKAP2L is higher in tumor tissues. **(F)** Paired differential analysis also means that tumor tissues in The Cancer Genome Atlas (TCGA) expressed more CKAP2L than the normal samples when the samples were collected from the same ccRCC patient. **** means *p*-value <.0001; *** means *p*-value ≥.0001 and <.001; ** means *p*-value ≥.001 and <.01; * means *p*-value ≥.01 and <.05; and ns means *p*-value≥ .05.

We first explored the expression level of CKAP2L in multiple tumoral tissues. The CKAP2L expression levels in tumor tissues and normal tissues were investigated using the UALCAN database. The results indicated that CKAP2L is more highly expressed in tumors than in normal tissues in bladder cancer (BLCA), breast cancer (BRCA), cervical squamous cell carcinoma and endocervical adenocarcinoma (CESC), cholangiocarcinoma (CHOL), colon adenocarcinoma (COAD), esophageal carcinoma (ESCA), glioblastoma multiforme (GBM), head and neck squamous cell carcinoma (HNSC), kidney chromophobe (KICH), KIRC, kidney renal papillary cell carcinoma (KIRP), liver hepatocellular carcinoma (LIHC), lung adenocarcinoma (LUAD), lung squamous cell carcinoma (LUSC), prostate adenocarcinoma (PRAD), pheochromocytoma and paraganglioma (PCPG), rectum adenocarcinoma (READ), sarcoma (SARC), stomach adenocarcinoma (STAD), and uterine corpus endometrial carcinoma (UCEC) (Figure 1A). In contrast to the UALCAN database, the Oncomine database includes comprehensive gene expression information from TCGA and other sources. We then explored CKAP2L expression in multiple kinds of tumor. The Oncomine database indicated that CKAP2L is highly expressed in bladder cancer, brain and central nervous system cancer, breast cancer, cervical cancer, colorectal cancer, gastric cancer, head and neck cancer, kidney cancer, liver cancer, lung cancer, lymphoma, melanoma, and ovarian cancer (Figure 1B). These results suggest that CKAP2L is highly expressed in diverse tumor tissues.

### CKAP2L is highly expressed in ccRCC tissues

**FIGURE 2:**
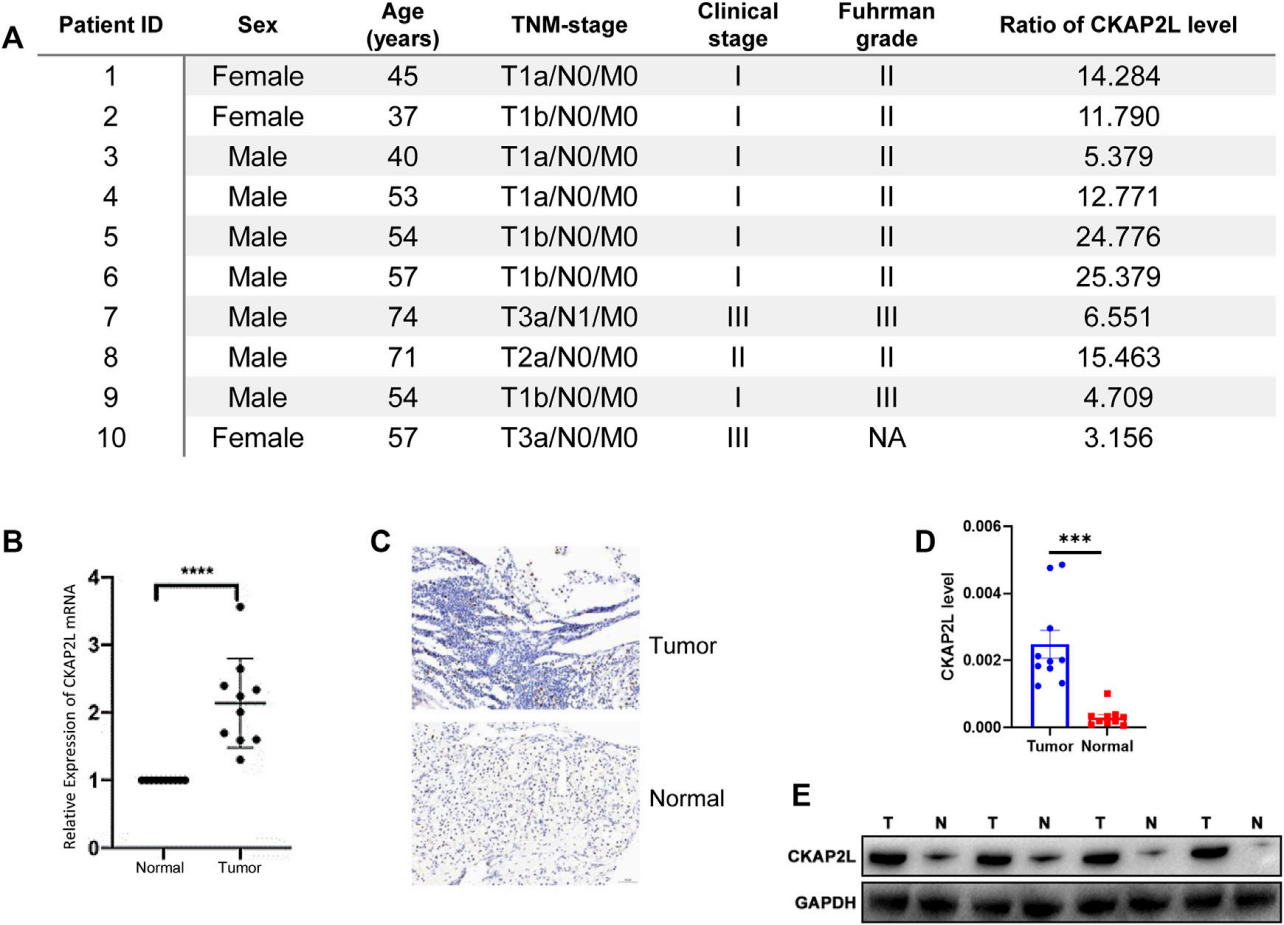
**(A)** Clinical characteristics and ratio of CKAP2L level in tumor and normal tissues in 10 ccRCC patients. **(B)** Real-time polymerase chain reaction (PCR) demonstrated high mRNA levels of CKAP2L in ccRCC tissues. **(C,D)** Immunohistochemical analyses were performed to detect differential CKAP2L in ccRCC and normal tissues at the protein level. **(E)** Western blot band shows the CKAP2L levels in tumor (T) and normal (N) tissues. *** means *p*-value ≥.0001 and <.001; ** means *p*-value ≥.001 and <.01; * means *p*-value ≥.01 and <.05; and ns means *p*-value≥ .05.

CKAP2L widely showed higher expression levels in human cancers according to the aforementioned analysis results. In some tumors, it has been validated by experiments but others, including ccRCC, are not. Therefore, it is necessary to evaluate the CKAP2L levels in ccRCC *via* public databases and experimental validations. Immediately, we then investigated the expression level of CKAP2L in ccRCC by analyzing the GEO database. We first examined the differential expression analysis results from several ccRCC tumor microarrays. The differential expression analysis results indicated that CKAP2L in tumor tissues is higher than in normal tissues in GSE76351, GSE53757, and GSE66270 (Figures 1C–E). A paired difference test is a statistical analysis method which could increase the statistical power or reduce confounding factors compared with the non-paired difference test. Differential expression analysis was performed on the TCGA ccRCC data, and the results showed that the paired differential expression analysis also indicated that CKAP2L is higher in ccRCC tissues (Figure 1F). In order to validate the experimental testing, 10 tumor and normal samples were collected from clinical patients after receiving approval from the ethical committee and consents of the patients. Their clinical characteristics are presented in Figure 2A. Subsequently, we investigated CKAP2L in the clinical ccRCC samples at both the protein and RNA levels. CKAP2L RNA levels between tumor and normal tissues were upregulated *via* RT-PCR detection (Figure 2B). Then, CKAP2L protein levels were detected *via* IHC (Figures 2C, D) and Western blot (Figure 2E), and the results strongly suggested higher CKAP2L levels in ccRCC tissues.

### CKAP2L level is associated with multiple ccRCC clinical characteristics and prognosis

**FIGURE 3:**
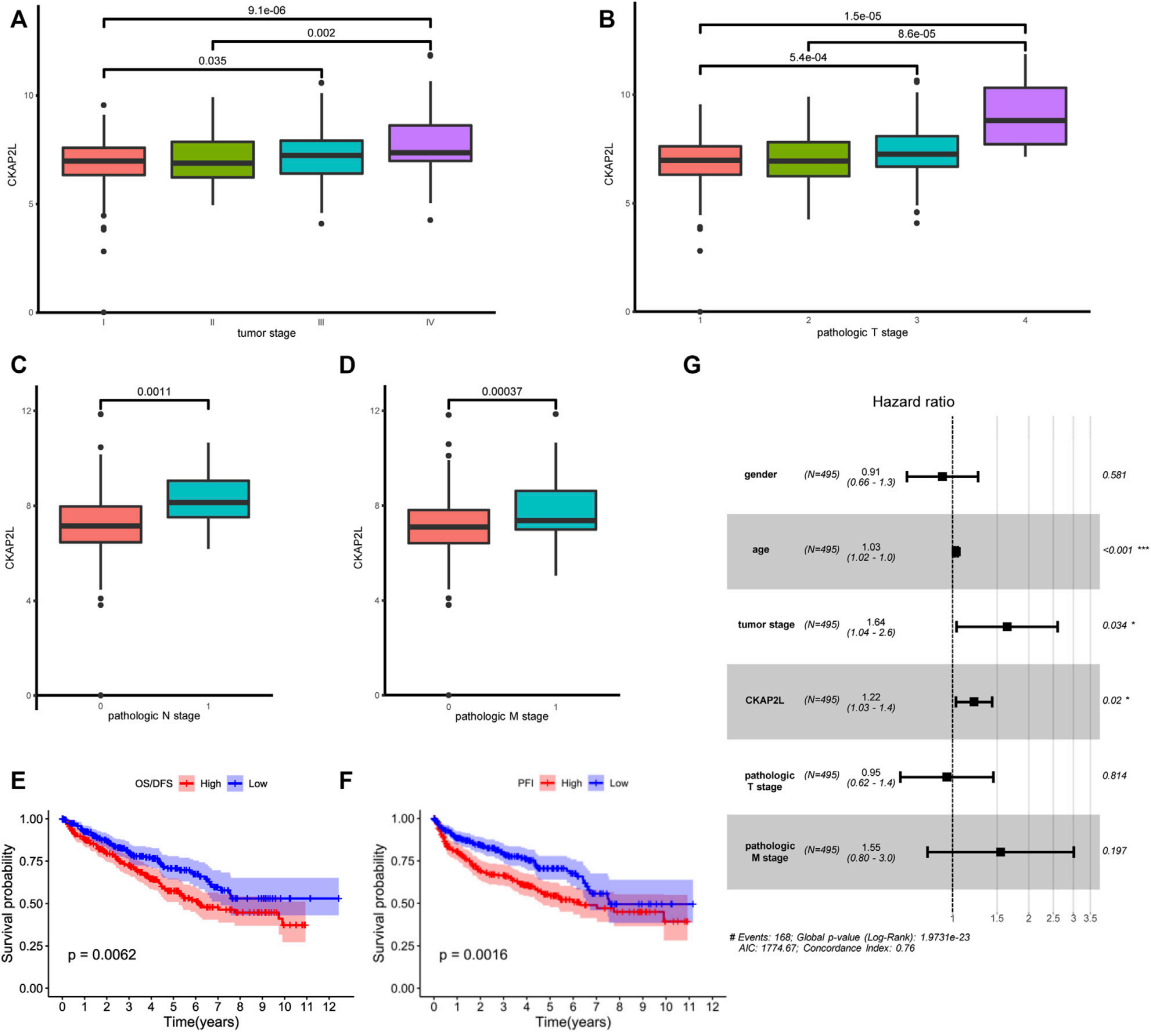
Relationship between cytoskeleton-associated protein 2-like protein (CKAP2L) and clinical characteristics. **(A)** Different CKAP2L levels at different clear cell renal cell carcinoma (ccRCC) stages. **(B–D)** Differential levels of CKAP2L in different ccRCC pathologic T, N, and M stages. **(E–F)** Kaplan–Meier survival curves revealed that ccRCC patients with higher or lower CKAP2L levels had different overall survival (OS) and disease-free survival (DFS). **(G)** Results of multivariate Cox regression indicated that CKAP2L is an independent prognostic factor in ccRCC patients. Adjusted *p*-value was labeled in the corresponding comparisons.

Subsequently, CKAP2L levels in ccRCC patients with different clinical stages were explored. We found that ccRCC patients with stages III and IV exhibited higher CKAP2L levels (Figure 3A). In pathologic T stages, T3 and T4 ccRCC patients expressed higher CKAP2L levels (Figure 3B), especially in the tumor T4 stage, where CKAP2L was significantly higher than in the other three T stages (Figure 3B). In pathologic N and M stages, we also found that the level of CKAP2L in N1 or M1 stage tumors was higher than that in the N0 or M0 stages, respectively (Figures 3C, D).

It is still unclear whether upregulated CKAP2L is associated with ccRCC outcomes. In this study, we first explored differences of the prognosis in ccRCC patients with different CKAP2L levels using the TCGA database. Patients were divided into two groups according to their CKAP2L levels, and survival analysis was performed. The results indicated that patients with higher CKAP2L have poorer OS/DSS and PFI (Figures 3E, F). We then explored prognostic variables from clinical features and CKAP2L level *via* univariate and multivariate Cox regression analysis. Univariate Cox analysis indicated that age, tumor stage, pathologic T and M stages, and CKAP2L level significantly influence the ccRCC prognosis (Table 1). Significant variables were subsequently analyzed by multivariate Cox analysis, and the results indicated that age, tumor stage, and CKAP2L level are independent prognostic factors for ccRCC patients (Figure 3G).

**TABLE 1:** Univariate Cox regression analyses for overall survival (OS) and disease-free survival (DFS) in ccRCC patients.

Variable	Hazard ratio (95% CI)	*p*-value
Gender	.6589–1.5174	.9996
Age at diagnosis	1.0034–1.0381	.0187
CKAP2L	1.1150–1.7050	.0030
Tumor stage	1.5314–2.2456	<.0001
Pathologic T stage	1.5000–2.4000	<.0001
Pathologic N stage	1.8773–6.6839	<.0001
Pathologic M stage	2.6889–6.4302	<.0001

### Potential pathogenic mechanisms of CKAP2L in ccRCC

**FIGURE 4:**
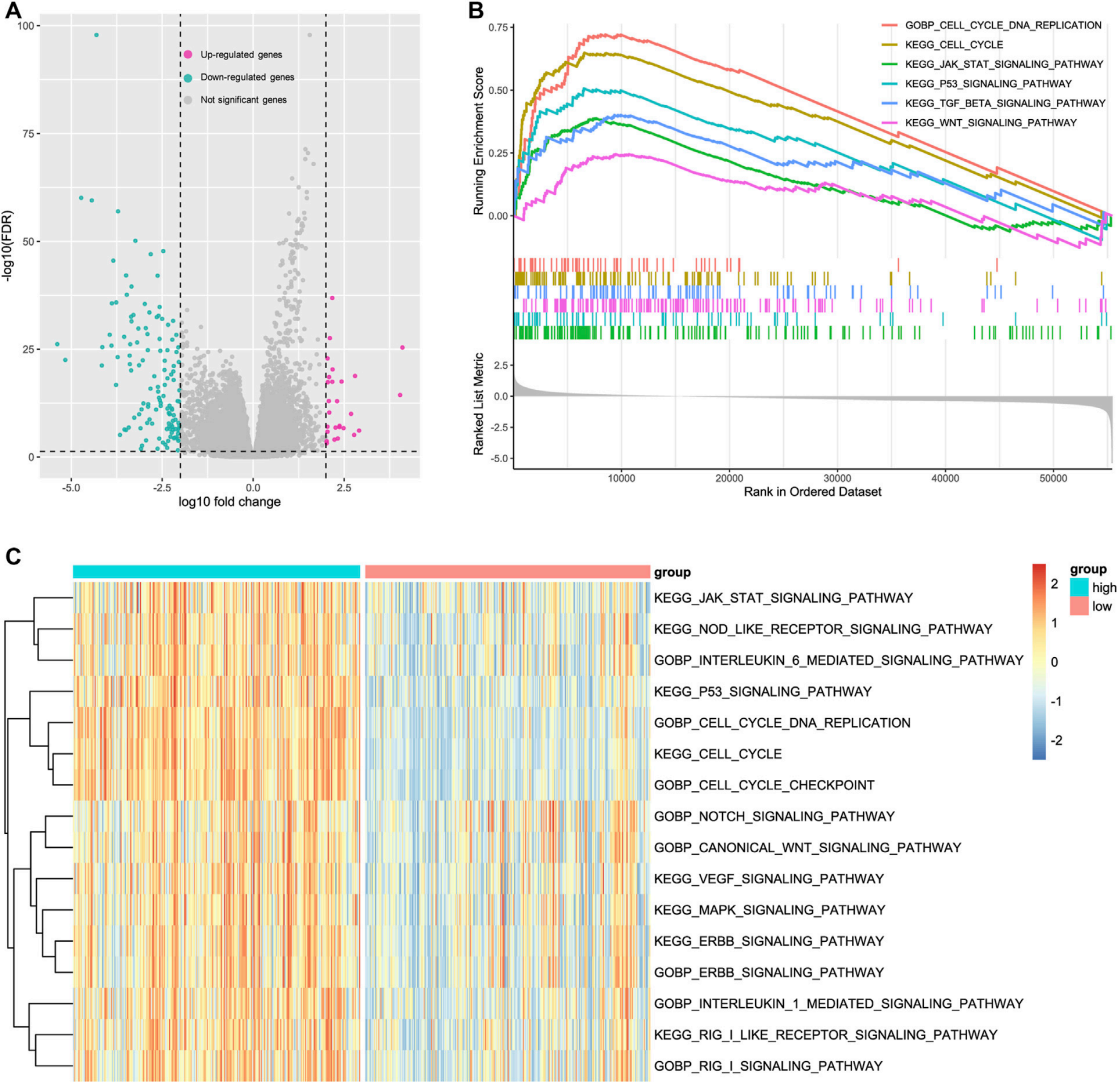
Potential mechanisms of cytoskeleton-associated protein 2-like protein (CKAP2L) in clear cell renal cell carcinoma (ccRCC) development. **(A)** Significantly up- and downregulated genes in ccRCC patients with high and low CKAP2L levels. Differentially expressed genes were defined as log10|fold change| > 2 and adjusted *p*-value (FDR) <.05. **(B)** Gene set enrichment analysis (GSEA) results revealed upregulated signaling pathways in patients with higher CKAP2L levels. All terms showed are satisfied adjusted *p*-value <.05. **(C)** Gene set variation analysis (GSVA) identified several activated and inactivated signaling pathways in patients with higher CKAP2L levels. All terms showed were compared by using the limma package and satisfied adjusted *p*-value <.05.

The aforementioned results indicated that CKAP2L is closely associated with ccRCC developments. However, little is known about how a high CKAP2L level promotes ccRCC. To further investigate the molecular biological functions of CKAP2L in ccRCC, TCGA ccRCC samples were divided into two groups according to CKAP2L level, and differential expression analysis was performed. A total of 27 upregulated genes and 129 downregulated genes were detected (Figure 4A). GSEA analysis was performed according to the differential analysis results. We found that the JAK–STAT signaling pathway, the P53 signaling pathway, the TGF-β signaling pathway, and the WNT signaling pathway are activated in higher-CKAP2L ccRCC samples (Figure 4B). GSVA is a method for estimating the variation of gene set enrichment by transforming the gene expression matrix to a gene set score matrix. Differential analysis at the pathway level can be achieved by combining GSVA with differential analysis. We found that samples with higher CKAP2L levels have higher JAK/STAT signaling pathway, interleukin (IL)-21 signaling pathway, NOTCH signaling pathway, MAPK signaling pathway, P53 signaling pathway, WNT signaling pathway, ERBB signaling pathway, IL-1 signaling pathway, and RIG I signaling pathway scores, which means those pathways are activated in higher-CKAP2L ccRCC tissues (Figure 4C). Furthermore, we noticed that cell division-related pathways were also activated in higher-CKAP2L ccRCC tissues, which suggests that CKAP2L may promote ccRCC by influencing the cell cycle and cell division (Figures 4B, C).

### Protein–protein interaction analysis of CKAP2L

**FIGURE 5:**
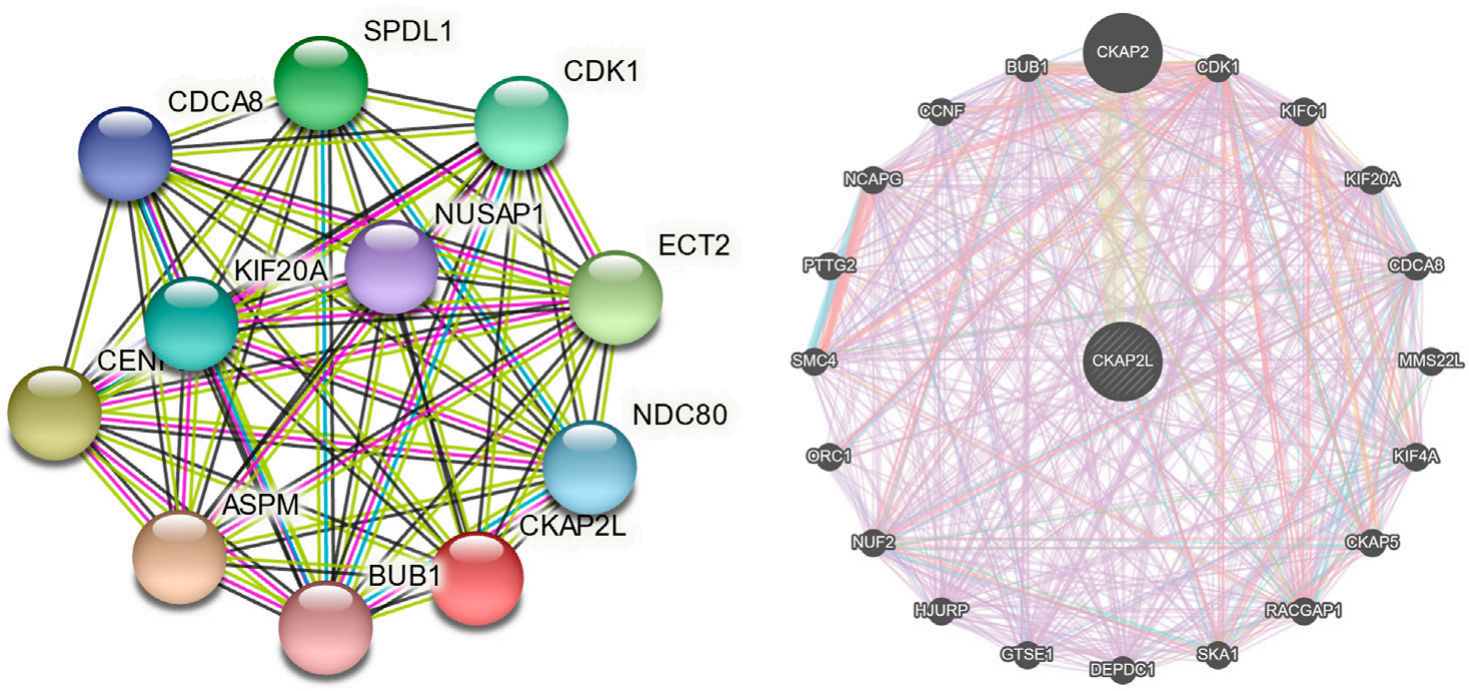
Protein–protein interaction networks of cytoskeleton-associated protein 2-like protein (CKAP2L) and other proteins. CKAP2L protein–protein interaction network from the Search Tool for the Retrieval of Interacting Genes/Proteins (STRING) database **(A)** and the GeneMANIA database **(B)**.

To further investigate the biologic functions of CKAP2L, STRING, and GeneMANIA, two databases that help explore gene interaction and functions were searched. The results from the two databases indicated that CAKP2L might interact with CDK1, CDCA8, BUB1, and KIF20A (Figures 5A, B). CDK1 is a cell cycle-related protein that is considered a novel therapeutic target in breast cancer. CDCA8 involves the regulation of mitosis and has been described as a potential prognostic biomarker in several cancers. BUB1, a mitotic checkpoint serine/threonine kinase, participates in the proliferation regulation of numerous tumors. KIF20A also takes part in multiple processes in cellular division. Protein–protein interaction analysis indicated that CKAP2L might influence tumor cell proliferation.

### Co-expression analysis of CKAP2L in ccRCC

**FIGURE 6:**
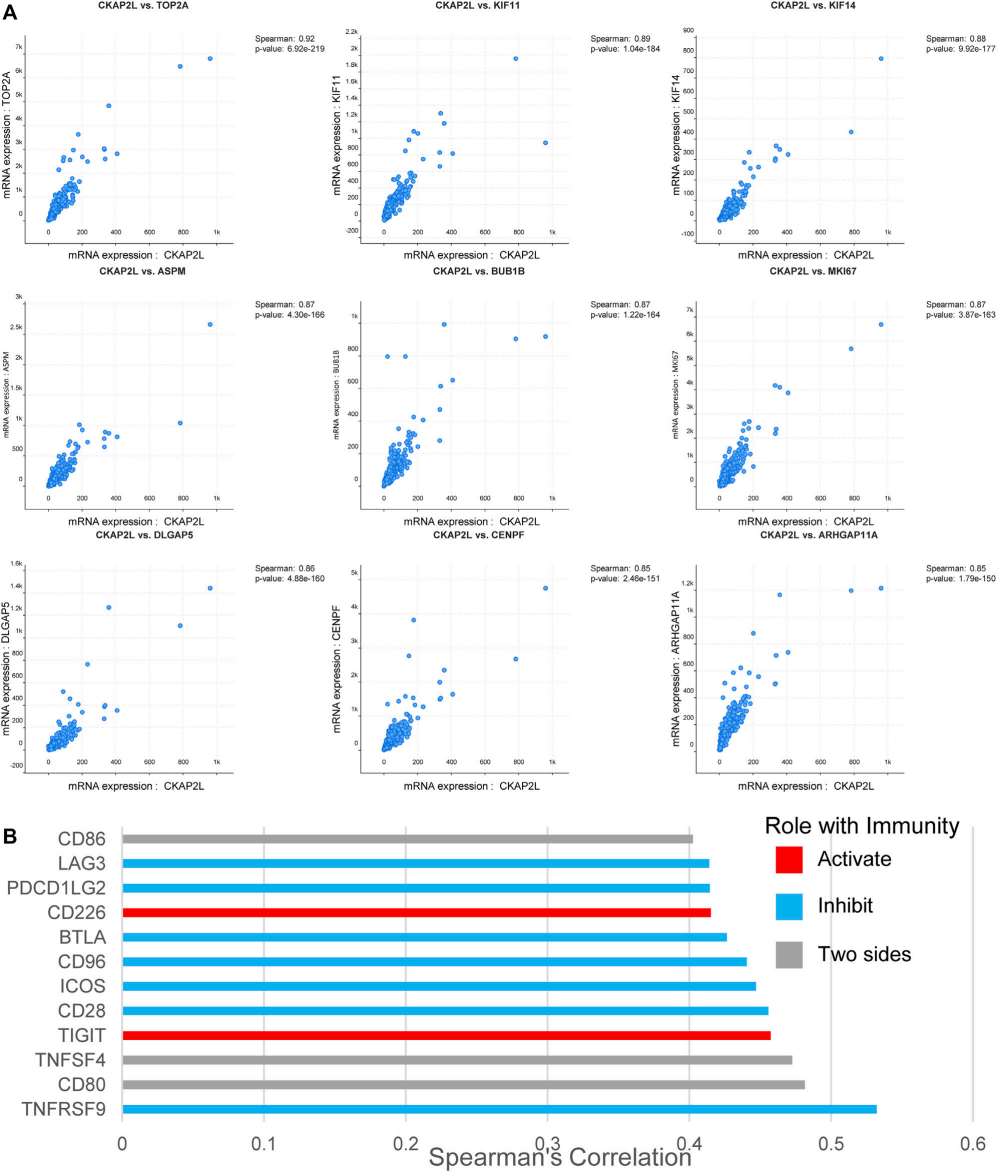
Cytoskeleton-associated protein 2-like protein (CKAP2L) co-expression in clear cell renal cell carcinoma (ccRCC) tissues. **(A)** Top nine CKAP2L co-expressed genes in ccRCC patients ranked by Spearman’s correlation coefficients. **(B)** CKAP2L is co-expressed with several immune checkpoint genes in ccRCC patients. All correlated immune checkpoint genes satisfied Spearman’s correlation coefficient >.4 and *p*-value <.05.

We explored the relationship between CKAP2L and other genes in the aspect of gene co-expression. The top nine and top 20 genes, ranked by Spearman’s correlation coefficients, are exhibited in Figure 6A and Table 2. CKAP2L is closely linked with cell cycle-related proteins TOP2A, BUB1B, MKI67, and DLGAP5. Subsequently, we investigated the relationship between CKAP2L and immune checkpoint genes. The results suggested that CKAP2L is significantly positively correlated with inhibitory immune checkpoint genes, such as ICOS, LAG3, BTLA, CD96, CD28, and TNFRSF9 (Figure 6B). However, CKAP2L is also positively correlated with two stimulatory immune checkpoint genes, CD226 and TIGIT (Figure 6B).

**TABLE 2:** Correlated expressional genes of CKAP2L in clear cell renal cell carcinoma (ccRCC) tissues.

Correlated gene	Spearman’s correlation	*p*-value	q-value
TOP2A	.920227	6.92 × 10^−219^	1.39 × 10^–214^
KIF11	.891126	1.04 × 10^–184^	1.04 × 10^–180^
KIF14	.882831	9.92 × 10^–177^	6.63 × 10^–173^
ASPM	.870704	4.30 × 10^–166^	2.15 × 10^–162^
BUB1B	.868947	1.22 × 10^–164^	4.88 × 10^–161^
MKI67	.867102	3.87 × 10^–163^	1.29 × 10^–159^
DLGAP5	.863202	4.88 × 10^–160^	1.40 × 10^–156^
CENPF	.85158	2.46 × 10^–151^	6.17 × 10^–148^
ARHGAP11A	.850374	1.79 × 10^–150^	3.97 × 10^–147^
SHCBP1	.850035	3.11 × 10^–150^	6.22 × 10^–147^
BUB1	.843232	1.57 × 10^–145^	2.86 × 10^–142^
DEPDC1	.842331	6.33 × 10^–145^	1.06 × 10^–141^
NEK2	.842066	9.52 × 10^–145^	1.47 × 10^–141^
EXO1	.839087	8.95 × 10^–143^	1.28 × 10^–139^
NCAPH	.838429	2.41 × 10^–142^	3.22 × 10^–139^
CEP55	.836371	5.21 × 10^–141^	6.52 × 10^–138^
NCAPG	.836224	6.47 × 10^–141^	7.62 × 10^–138^
ESCO2	.835853	1.12 × 10^–140^	1.25 × 10^–137^
SGO1	.831333	8.03 × 10^–138^	8.47 × 10^–135^
HMMR	.828348	5.55 × 10^–136^	5.56 × 10^–133^

### CKAP2L level may be related with specific immune cell infiltration and influence prognoses

**FIGURE 7:**
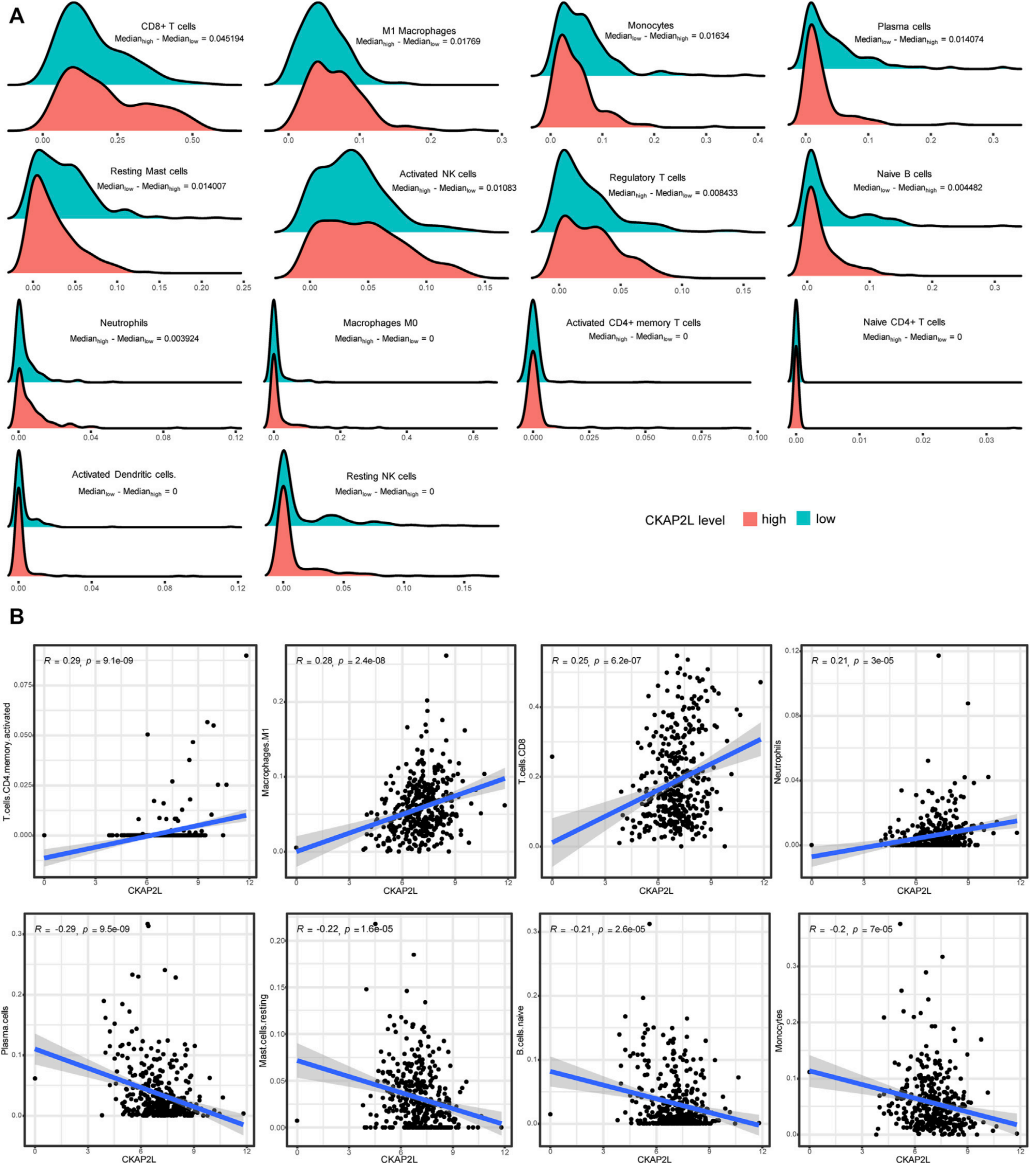
Relationships of cytoskeleton-associated protein 2-like protein (CKAP2L) and immune infiltration in clear cell renal cell carcinoma (ccRCC). **(A)** Differences in the level of several immune cells in higher- and lower-CKAP2L ccRCC tissues. **(B)** Relevance of CKAP2L and several immune cell levels in ccRCC tissues. Spearman’s correlation coefficient and *p*-value are shown in the top right of scatter plots.

According to the aforementioned analysis, we noticed that CKAP2L is associated with several immune checkpoint genes, which suggested that CKAP2L may be related with specific immune cell infiltration patterns. Thus, we investigated the relationship between CKAP2L and immune microenvironments in ccRCC. CIBERSORT was chosen to estimate the immune cell score in each ccRCC sample by analyzing TGCA databases. The relative contents of immune cells were compared between higher-CKAP2L and lower-CKAP2L groups. It was found that scores were elevated for CD8^+^ T cells, naïve CD4^+^ T cells, activated memory CD4^+^ T cells, Tregs, activated NK cells, M0 macrophages, M1 macrophages, and neutrophils and depressed for naïve B cells, plasma cells, resting NK cells, monocytes, activated dendritic cells (DCs), and resting mast cells (Figure 7A). Correlation analysis indicated that the CKAP2L level was positively correlated with activated CD4^+^ memory T cell, M1 macrophage, CD8^+^ T cell, and neutrophil scores and negatively correlated with plasma cell, resting mast cell, naïve B cell, and monocyte scores (Figure 7B). However, it is notable that the difference between the higher- and lower-CKAP2L groups of immune cell-relative contents is more obvious in CD8^+^ T cells, M1 macrophages, monocytes, and plasma cells, but not in other cells, such as activated DCs, resting NK cells, and naïve CD4^+^ T cells (Figure 7A).

**FIGURE 8:**
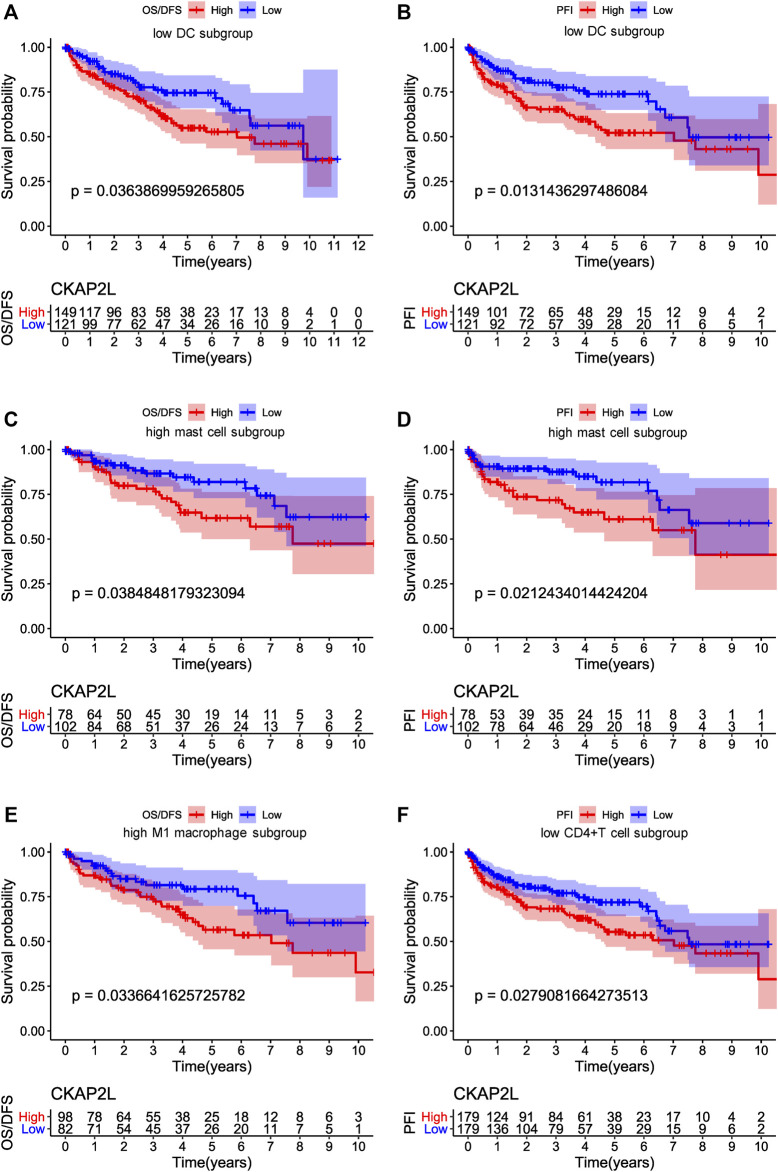
Influences of immune cell and cytoskeleton-associated protein 2-like protein (CKAP2L) levels on overall survival (OS), disease-free survival (DFS), and progression-free interval (PFI) in clear cell renal cell carcinoma (ccRCC) patients. **(A,B)** represent the OS/DFS and PFI, respectively, of ccRCC patients with a lower proportion of dendritic cells. **(C,D)** represent the OS/DFS and PFI, respectively, of ccRCC patients with a high proportion of mast cells, and **(E,F)** represent the OS/DFS and PFI, respectively, of ccRCC patients with a high proportion of M1 macrophages.

We would like to know whether CKAP2L impacts the prognosis of ccRCC patients with specific immune infiltration characteristics. Survival analysis was performed on higher and lower relative-immune cell ccRCC patients. The results suggest that lower-CAKP2L patients with lower DCs and CD4^+^ T cells or higher mast cells and M1 macrophages have more poor survival outcomes (Figures 8A–F).

**FIGURE 9:**
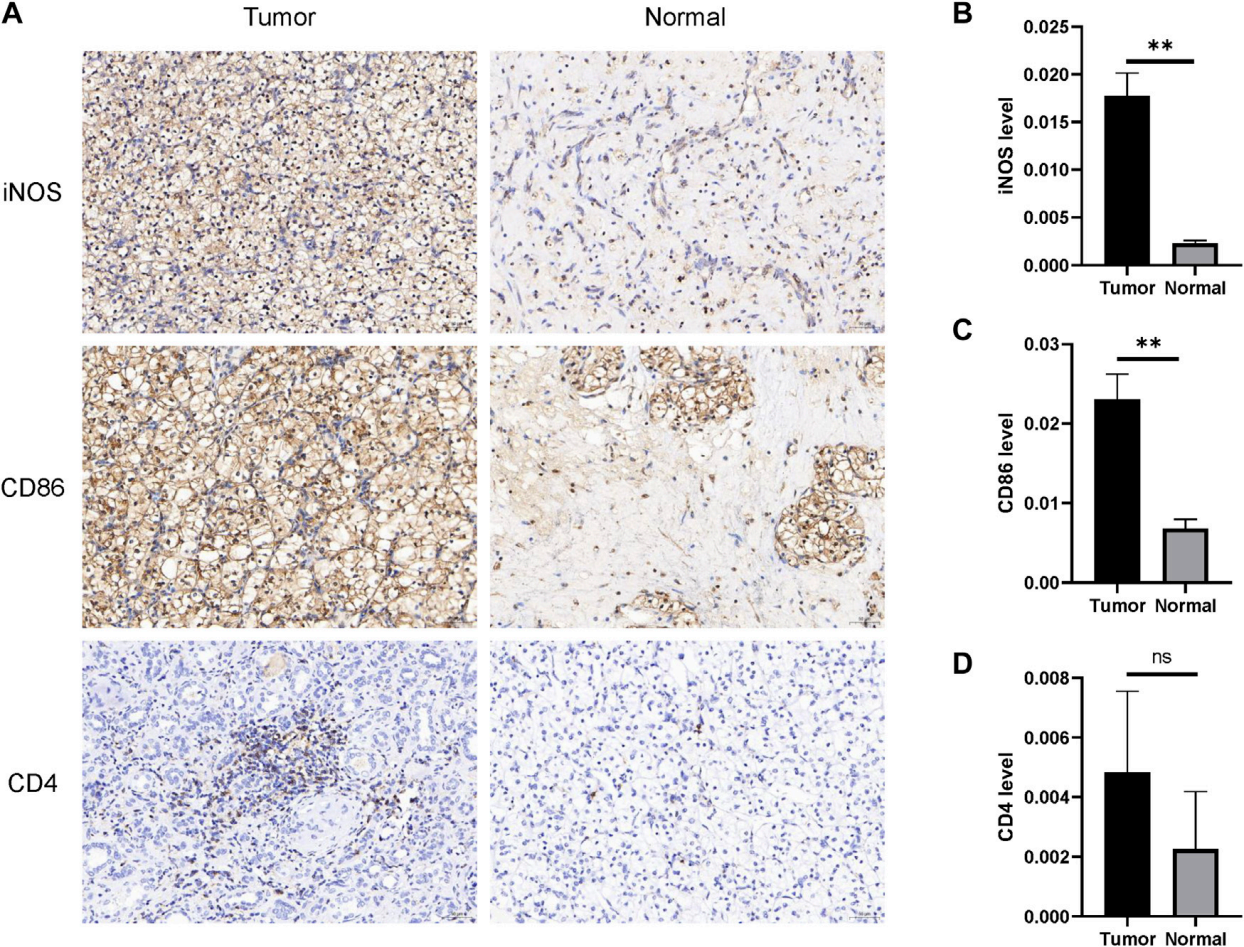
IHC validates M1 macrophage infiltrations in tumor tissues with higher CKAP2L. **(A)** M1 macrophage (iNOS and CD86) and CD4^+^ T cell (CD4) infiltrations in tumor tissues with higher CKAP2L in tumor tissues and lower CKAP2L in normal tissues. **(B–D)** Contrast differences of iNOS, CD86, and CD4 between tumor and normal tissues. ** means *p*-value ≥.001 and <.01; * means *p*-value ≥.01 and <.05; and ns means *p*-value≥ .05.

Although bioinformatics analysis suggested that higher-CKAP2L ccRCC tissues are associated with specific immune microenvironments including higher M1 macrophages. Moreover, specific immune microenvironments, like lower CD4^+^ T cell may be related to worse prognosis. It is useful for future as ccRCC improves prognosis prediction. Nevertheless, they are short of experimental validation. In this study, the difference of the M1 macrophage level between tumor and normal tissues was examined by IHC. We found that there are higher M1 macrophage markers (CD86 and iNOS) in tumor tissues than in normal tissues, accompanied with higher CKAP2L levels in tumor tissues (Figures 9A–C). Although the total CD4^+^ T cell level was not correlated with the CKAP2L level, patients with lower CD4^+^ T cell and higher CKAP2L have poor prognosis. We then examined the CD4 levels between higher-CKAP2L tumor tissues and lower-CKAP2L normal tissues. However, CD4^+^ levels had no differences between them (Figures 9A, D).

## Discussion

ccRCC is one of the most widespread malignant tumors across the globe (Choueiri et al., 2021). With regard to assessing the prognosis in ccRCC patients, the TNM staging system is widely used in clinical procedures (Nakayama et al., 2014); however, these predictive frameworks are not always useful indicators for undertaking the prognosis in ccRCC victims (Qin et al., 2013). The progression of ccRCC in patients is monitored by analyzing those molecules that possess tumorigenic properties (Quan et al., 2021).

In the past few years, several studies have provided evidence that CKAP2L expression patterns are linked with physiological and pathological processes (Li et al., 2020; Feng et al., 2021; Monteverde et al., 2021; Zhang et al., 2021). CKAP2, an important paralog of CKAP2L, participates in the pathophysiological process, although it also causes initiation and growth of some tumors (Ippolito et al., 2016; Zhang et al., 2018). The current evidence indicates that CKAP2 is not likely to be a favorable prognostic factor in glioma, breast cancer, and ovarian cancer (Sim et al., 2017; Zhang and Zhao, 2017; Wang et al., 2018). Furthermore, it has been suggested by work on molecular mechanisms that the proliferation and motility of cancer cells are usually controlled by various CKAP2-mediated signaling pathways. Xiong et al. suggested that CKAP2L might promote cell proliferation partially by regulating the MAPK signaling pathway (Xiong et al., 2019). Li et al. reported that CKAP2L knockdown inhibits glioma cell proliferation, migration, invasion, and epithelial–mesenchymal transition (Li et al., 2020). CKAP2L knockdown also induced cell cycle arrest at the G2/M phase (Li et al., 2020). Thus, our hypothesis is that CKAP2L may promote ccRCC progression.

In this study, we found that CKAP2L is upregulated in plenty of tumors according to a public database. Then, we focused on the expression level of CKAP2L in ccRCC, from the aspect of mRNA and proteins, by exploring public databases and detecting clinical samples, and the results showed that CKAP2L is upregulated both in RNA level and protein level. Subsequently, we found that CKAP2L expression level is related to several crucial clinical characteristics, such as tumor stage and TNM stage in ccRCC patients. Survival analysis and Cox regression analysis indicated that CAKP2L is closely related to the prognoses of ccRCC patients.

It is necessary to explore the potential molecular mechanisms of CKAP2L in promoting ccRCC developments. Gene enrichment analysis, including GSEA and GSVA, finds that CKAP2L might boost ccRCC by activating a series of vital tumor-related pathways, such as the JAK–STAT signaling pathway, the P53 signaling pathway, and the TGF-β signaling pathway. Moreover, results of gene enrichment analysis, protein–protein interaction analysis, and co-expression analysis suggest that CKAP2L might affect the cell cycle and cellular division in ccRCC.

There is a subtle relationship between CKAP2L and immune microenvironments in ccRCC. First, CKAP2L is positively correlated with numerous inhibitory immune checkpoint genes. This indicates that CKAP2L might mainly influence immune system functions indirectly, which eventually boosts ccRCC development, although CKAP2L is also positively correlated with two stimulatory immune checkpoint genes. Lots of immune-cell subtypes, such as activated CD4^+^ memory T cells, M1 macrophages, CD8^+^ T cells, and neutrophils, showed their close relationships in correlation analysis. However, survival analysis indicated that only DCs, CD4^+^ T cells, mast cells, and M1 macrophage levels might affect ccRCC prognoses *via* CKAP2L. IHC of small sample ccRCC tissue sections primarily validated that higher CKAP2L may be accompanied with higher M1 macrophages, but there is no relationship between CKAP2L and CD4^+^ T cells. Our results remind us that CKAP2L might exert different functions in different immune microenvironments. The immunological abnormalities related to CKAP2L level in ccRCC should be further explored and validated in future large cohort studies.

## Conclusion

In summary, our study illustrates that CKAP2L is upregulated in ccRCC, which promotes ccRCC development and worsens the ccRCC prognosis. Furthermore, we find that CKAP2L might exert biologic effects in ccRCC by affecting the cell cycle and activating specific tumor-related signaling pathways. The study also suggests that CKAP2L is related to specific tumor immune microenvironments. Through this statement, it should be noted that the majority study conclusions of CKAP2L in ccRCC are based on bioinformatics analysis, and only a very small part has been examined by small sample tumor tissues. CKAP2L could be a potential prognostic biomarker and molecular treatment target for ccRCC. In future, perspective clinical studies with larger sample sizes should be performed to fully validate the association between CKAP2L and immune signatures and prognosis. More rescue experiments should be implemented to explore the biological functions of CKAP2L in ccRCC cells.

## Data Availability

Publicly available datasets were analyzed in this study. These data can be found here: all relevant raw data used in the study can be accessed from TCGA (https://portal.gdc.cancer.gov/), GEO (https://www.ncbi.nlm.nih.gov/geo/), UALCAN (http://ualcan.path.uab.edu/), Oncomine (https://www.oncomine.com/), and UCSC-XENA (https://xena.ucsc.edu/).
